# The Presence and Size of the Corpus Luteum Influence the In Vitro Production of Sheep Embryos

**DOI:** 10.3390/vetsci12080690

**Published:** 2025-07-24

**Authors:** Alfredo Lorenzo-Torres, Raymundo Rangel-Santos, Yuri Viridiana Bautista-Pérez, Juan González-Maldonado

**Affiliations:** 1 Departamento de Zootecnia, Universidad Autónoma Chapingo, Texcoco 56230, Mexico; rangelsr@correo.chapingo.mx (R.R.-S.); al18102352@chapingo.mx (Y.V.B.-P.); 2 Instituto de Ciencias Agrícolas, Universidad Autónoma de Baja California, Mexicali 21705, Mexico; juan.gonzalez.maldonado@uabc.edu.mx

**Keywords:** oocyte, embryos, corpus luteum, in vitro production, ovaries

## Abstract

The corpus luteum (CL) is a transient gland that can directly influence follicular dynamics and oocyte quality. The objective of this study was to evaluate the influence of the absence or presence of a small (≤3 mm), medium (4–8 mm), or large (>8 mm) CL in slaughterhouse ovaries on in vitro embryo production. Cumulus–oocyte complexes (COCs) were collected from each group of ovaries and matured in TCM-199 medium, plus hormones and fetal bovine serum. Fertilization was performed with fresh semen from a Katahdin ram of known fertility. Embryo development was carried out in commercial sequential media for 72 and 96 h, until the blastocyst stage. The number of follicles (2–6 mm in diameter) and COCs were influenced by the presence of CL, which was higher (*p* < 0.05) in the Large CL group (5.51 ± 0.33 and 3.62 ± 0.27) compared to the Without CL group (4.54 ± 0.19 and 2.62 ± 0.14, respectively), with no difference between the CL sizes. Likewise, the diameter and area of the COCs were higher in the Small CL group of ovaries compared to the Without CL group. In the Large CL group of ovaries, 9% more morulae (*p* < 0.05) were obtained compared to the Without CL group; in the Medium CL group, 13% more blastocysts were obtained compared to the Without CL group. However, in the hatching capacity and diameter of blastocysts, no statistical difference was evident (*p* > 0.05). In conclusion, the presence and size of the CL in the ovaries of slaughtered sheep influence the productive efficiency of embryos in vitro under the conditions in which the present study was carried out.

## Simple Summary

The corpus luteum (CL) is a transient gland that influences follicular development, oocyte quality, and subsequent development. This study evaluated the absence or presence of a small (≤3), medium (4–8), or large (>8 mm) CL in the ovaries of slaughtered sheep on in vitro embryo production. Cumulus–oocyte complexes (COCs) were collected, matured, and placed in a CO_2_ incubator. Subsequently, fertilization was performed with fresh semen from a ram of known fertility. Zygotes were cultured in sequential media to evaluate blastocyst hatchability. In the Large CL group, the number of follicles and COCs was higher compared to the No CL group. In addition, among the most notable results, it was better in the CL groups. This is important because the presence of the CL can be included as a strategy in ovarian selection to obtain oocytes with greater in vitro development capacity.

## 1. Introduction

Oocyte competence is crucial for optimal in vitro embryo development, and it influences the establishment and maintenance of pregnancy [1]. The efficiency of in vitro-produced embryos is likely more closely related to the oocyte source than to culture conditions [2]. In this regard, the presence of ovarian structures, such as preovulatory follicles or corpora lutea, influences the competence of the collected oocytes [3,4,5]. The CL is a transient endocrine gland that forms from the Graff follicle after oocyte release, through morphological and biochemical changes. It secretes progesterone (P4) as the main steroid hormone [6]. There is extensive information about the influence of the CL on oocyte quality [5,7,8,9,10]. However, the results are contradictory because the CL is reported to either positively or negatively affect oocyte viability [4,11,12]. The presence of the CL has been shown to favor oocyte metabolic activity and is associated with enhanced embryo development [13]. There is also evidence of positive effects from the high concentration of P4 due to the presence of the CL [8], improving the competence of oocytes ipsilateral to this structure [14], through the P4 diffusion to nearby follicles [12]. Furthermore, it has been reported that the presence of P4 could have anti-apoptotic effects on the cumulus–oocyte complex [15], which could be reflected in its subsequent development. In addition, it has been suggested that ovaries with CL have higher vascularization and allow a high flow of nutrients and hormones than those without CL [16], supporting follicular development. Greater metabolic activity measured by the activity of the G6PDH enzyme has been reported in oocytes from ovaries with CL, in addition to greater production of blastocysts (68.2%) than in ovaries without CL (38.7%) [13]. However, some authors have not found favorable effects from the presence of the CL, and have even reported negative effects [11]. Azari-Dolatabad et al. [12] reported lower development and quality of in vitro-produced bovine embryos, with a threefold higher E2/P4 ratio in follicular fluid from ovaries without CL compared to ovaries with CL. The authors report that when the CL is present, the P4 concentration in follicular fluid is higher (134.1 ng mL^−1^) than when the CL is not present in the ovaries (86.0 ng mL^−1^). On the other hand, high CL activity with high P4 production in cows improves embryo quality and production [5]. Thus, in addition to the presence of the CL, its size could influence oocyte competition and embryo production, because the luteal vascular growth rate is higher in mature CLs [6], and this is related to the concentration of P4 [17]. Contreras-Solís et al. [18] reported a correlation between P4 concentration and CL diameter and area, with *r* = 0.599 and *r* = 0.565, respectively. They also mentioned that plasma P4 concentration increased from day 1 to day 11 of the estrous cycle, with luteal tissue being functional from day 3 to day -2 of the cycle (>0.5 ng/mL). Unlike in cattle, there is little information regarding the influence of CL and its size on in vitro embryo production in sheep. Therefore, the objective of this study was to evaluate the influence of the absence or presence of a small, medium, or large CL in the ovaries of slaughterhouse sheep on the number of antral follicles and the number and size of collected COCs, as well as this influence on in vitro embryo production efficiency.

## 2. Materials and Methods

### 2.1. Ethics Statement

The ovaries were collected from a commercial slaughterhouse according to the specifications of the Mexican official standard NOM-033-ZOO-1995, Humane Killing of Domestic and Wild Animals [19]. Mixed individual ovaries (*n* = 332) were collected from 2- to 3-year-old, 60 to 70 kg, non-pregnant, hormonally unstimulated, and clinically healthy hair sheep during the breeding season. This information was provided by the slaughterhouse operator and corroborated with the records. The ovaries were transported in a thermos with saline solution (0.9% NaCl) supplemented with 50 µg of gentamicin sulfate at 37 °C, within the first 3 h after the sheep were slaughtered.

### 2.2. COC Recovery

The slaughterhouse ovaries from five replicates were washed three times in isothermal saline (0.9% NaCl, 35 °C) upon arrival at the laboratory. Before puncture, the number of antral follicles present in four groups of ovaries was recorded: Without CL and Small, Medium, and Large CL. Immediately after, the COCs were collected using a 10 mL syringe and a 21 G needle (18 × 38 mm). The recorded and punctured follicles were those 2 to 6 mm in diameter, measure with a vernier caliper. The medium used for follicular puncture was TCM-199 with Hepes plus 50 µg mL^−1^ gentamicin sulfate and 50 IU heparin mL^−1^ [20]. Only COCs with intact cumulus and homogeneous cytoplasm [21] were selected for the present experiment, using a stereomicroscope (Leica S8APO; Leica Microsystems, Wetzlar, Germany) and recording the number of structures for each group. The diameters and areas of the COCs were measured using digital camera software version 3.7 (AmScope, Irvine, CA, USA). The measurement was made considering that the entire COC was within the circle drawn along the perpendicular diameter lines.

### 2.3. Maturation, Fertilization, and In Vitro Embryo Development

The COCs collected from each group of ovaries were washed and matured in independent wells (Nunc, Roskilde, Denmark), which contained TCM-199 medium with Hepes, supplemented with 10% fetal bovine serum (*v*/*v*) (Biowest, Mayimex S.A., Mexico City, Mexico), 5 μg mL^−1^ FSH (Folltropin-V, Bioniche, Ontario, Canada), 5 IU mL^−1^ hCG (Chorulon; Merck, NJ, USA), and 1 μg mL^−1^ 17-β estradiol (Estradiol, Sigma Aldrich, Mexico City, Mexico) for 24 h [22]. The incubation conditions for the entire experiment were 5% CO_2_, 38.5 °C, and saturated humidity (CB 170, Binder, Germany). Fertilization was performed with fresh semen from a Katahdin ram of known fertility, collected using an artificial vagina. After collection, the semen was kept in the dark at 22 °C for 90 min [23]. A 100 µL aliquot of pure semen was used for sperm capacitation. The semen was washed and centrifuged at 225× *g* for 3 min. Final capacitation was performed using the swim-up technique [24], placing a fraction of the semen sedimented during centrifugation at the bottom of the column with a sperm capacitation medium (In vitro S.A., Mexico City, Mexico). After 5 min of semen incubation, the upper column fraction was collected and used for in vitro fertilization. The matured COCs were incubated with capacitated sperm for 22 h, using a concentration of 1 × 10^6^ mL^−1^ of spermatozoa. After this time, the putative zygotes were washed and gently stripped with a micropipette. Zygotes were cultured in groups of 30 to 40 in commercial cleavage medium (Cook IVF, Brisbane, Australia) and mineral oil for 72 h. After this time, the number of embryos with at least 16 cells was counted, and these were considered morula embryos. These embryos were subsequently transferred to a commercial blastocyst medium (Cook IVF, Brisbane, Australia) plus mineral oil, where they remained for 96 h until they reached the blastocyst stage. The number of blastocysts was determined at the end of culture by evaluating their morphology [25] under an inverted microscope (Nikon Eclipse TS100; Nikon, Tokyo, Japan). A healthy blastocyst was defined as an embryo that showed a clear differentiation between the dark, compact inner cell mass and the trophectoderm, formation of the blastocoel, and an intact zona pellucida. Once identified, the diameter of the blastocysts produced was measured with the aid of a digital camera adapted to an inverted microscope (×200). The average diameter from two perpendicular measurements, including the zona pellucida, was recorded. To determine hatchability, the blastocysts were placed back into the culture medium for 24 h. After this period, the number of blastocysts that ruptured the zona pellucida and expelled at least 50% of their cellular contents or were fully hatched was recorded.

### 2.4. Experimental Design

In the present study, four experimental groups were evaluated to determine the influence of the absence or presence of the CL in the ovaries of slaughtered sheep on the number of follicles, the number and size of the collected COCs, and their capacity for embryo development in vitro. The groups were G1 = ovaries without CL; G2 = ovaries with small CL (≤3 mm); G3 = ovaries with medium CL (4–8 mm), and G4 = ovaries with large CL (>8 mm in diameter).

### 2.5. Statistical Analysis

Data for the study variables were analyzed using SAS software (version 9.1.3) [26] (SAS, Cary, NC, USA, 2012), following the guidelines of Gbur et al. [27]. The variables number of follicles and number of COCs collected were analyzed using the GLIMMIX procedure, considering a Poisson distribution and the Logit function. The variables number of morulae, blastocysts, and hatching capacity were analyzed using the same procedure, but considering a binomial distribution and the Logit link function. Finally, the variables COC diameter and area, as well as blastocyst diameter, were analyzed as normally distributed variables using the GLM procedure. The model used for these variables was as follows:

$$y_{ij}=\mu +T_{i}+E_{ij}$$

where $y_{ij}$ = diameter and area of the COC or diameter of the blastocyst of the *j*th repetition and of the *i*th group with or without CL (Small, Medium, and Large); μ = population mean; $T_{i}$ = fixed effect of the *i*th group with or without CL (Small, Medium, and Large); and $E_{ij}$ = random effect of the experimental error of the variables assuming an identical and independent normal distribution with zero mean and variance (${\sigma^{2}}_{e}$). The results were reported as least-square means ± standard error (LSM ± SE) for all variables, and the ESTIMATE option was used for the comparison of means, considering a *p* < 0.05.

## 3. Results

### Number of Follicles and COC Collection

**Figure 1:**
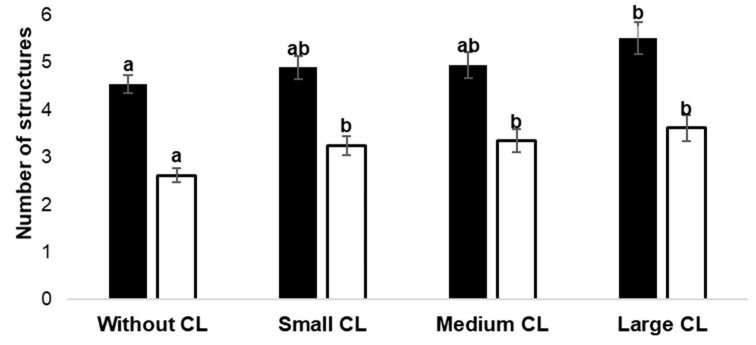
Influence of the absence or presence of Small (≤3 mm), Medium (4–8 mm), or Large (>8 mm) CL in the ovaries of slaughterhouse sheep on the number of follicles (black column) and the number of cumulus–oocyte complexes (COCs) collected (white column). Each bar represents the least-squares mean ± standard error. Different letters (a and b) between columns of the same color represent statistical differences (*p* < 0.05).

A total of 995 COCs were collected from 332 ovaries of healthy slaughtered ewes, resulting in a recovery efficiency of 61.4% (1620 follicles/995 COCs). The number of COCs collected in each group was as follows: Without CL = 339, Small CL = 274, Medium CL = 197, and Large CL = 185. The number of aspirated follicles was influenced (*p* < 0.05) by the ovarian status of the experimental groups: Without CL (4.54 ± 0.19), Small CL (4.89 ± 0.24), Medium CL (4.94 ± 0.28), and Large CL (5.51 ± 0.33) (Figure 1). The Large CL group of ovaries showed a higher (*p* = 0.008) number of follicles than the Without CL group of ovaries.

A lower number of COCs was collected (2.62 ± 0.14) in the group of ovaries Without CL compared to the groups with Small (3.25 ± 0.19), Medium (3.34 ± 0.24), and Large CL (3.62 ± 0.27), with no difference (*p* > 0.05) between CL sizes. On the other hand, the diameter and area of the COCs were higher in the group of ovaries with Small CL compared to the group Without CL (*p*< 0.05) (Table 1).

The COCs underwent maturation, fertilization, and embryo culture, with an overall percentage of 83.4% of the embryos reaching the morula stage. The Large CL ovary group showed 9% more (*p* = 0.05) morula production than the Without CL ovary group, but similar (*p* > 0.05) production compared to the other CL groups (Table 2).

The Medium CL group produced 13% more blastocysts (*p* = 0.04) compared to the Without CL group (Table 3). Furthermore, the Large CL group tended to be different from the Without CL group (*p* = 0.08). In the case of hatched blastocysts, no differences were evident, with an average hatching rate of 21.6%. In addition, no differences in blastocyst diameter (*p* > 0.05) were observed between the groups (Table 4). The average diameter of sheep embryos was 208.43 µm.

## 4. Discussion

Ovarian status is one of the most important factors influencing oocyte competence and subsequent embryo development. In the present study, the influence of the absence or presence of a small, medium, and large CL in the ovaries of healthy slaughtered sheep was evaluated on variables associated with in vitro embryo production. The results showed a higher number of follicles, higher number and larger size of COCs, and higher production of morula-stage embryos and blastocysts in the Large CL group of ovaries compared to the Without CL group of ovaries. No differences were found in hatching capacity or blastocyst diameter. This is relevant because oocyte competition appears to be high when the CL with functional characteristics is present in sheep ovaries.

In the Large CL group, almost one additional aspirated follicle (2–6 mm) was obtained compared to the Without CL group of ovaries. Furthermore, it was observed that the presence of the CL, regardless of size, produced a greater number of excellent-quality oocytes (≥3.25). In addition, the presence of small CL produced COCs with a larger diameter and area compared to the Without CL group. This could be related to the number of granulosa cell layers, which is associated with higher oocyte quality [21]. There are contradictory results in the literature regarding the influence of the CL on oocyte competence. Some studies have reported that the presence of the CL has adverse effects on the quality and production of in vitro embryos [11,12]. However, the results of the present study support the positive effect of the presence of the CL at the time of oocyte collection.

Similar results were reported in cattle, where a higher number of grade 1 COCs were obtained from ovaries with CL compared to the group of ovaries without this structure [4], although the authors found no association between CL and excellent-quality oocytes. Furthermore, it has been shown that the diameter of oocytes collected from slaughterhouse ovaries with CL was 2.4 to 3 µm greater than that of oocytes collected from ovaries without CL, the ZP was thinner, and metabolically, they appeared to be more developed oocytes due to the activity of G6PDH [13]. This is relevant because if oocyte quality is better in the presence of the CL, it could be considered an ovary selection method based on ovarian structures from the estrous cycle, providing a non-invasive criterion for accessing competent oocytes [28]. In the present study, oocyte competence after maturation was not directly assessed; rather, their ability to produce viable in vitro embryos was evaluated.

In the case of in vitro embryo production, 88% of morulae were produced in the Large CL group versus 78% in the Without CL group, with no differences between CL size categories. Likewise, in the case of blastocysts, the percentage was higher in the Medium CL group at 13% compared to the Without CL group. These results coincide with those reported in cows; Argudo et al. [13] found that 35.6% more blastocysts were produced in the oocytes collected from ovaries with CL than in the group of contralateral ovaries without CL. Similarly, inducing the persistence of the CL during hormonal treatment in superovulated sheep favored the production of transferable embryos compared to the group without prior knowledge of the ovarian status (7.4 vs. 4.1) [29]. Furthermore, the authors showed that oocytes collected from ovaries with CL in superovulated sheep showed better in vitro development to the blastocyst stage (35.8%) compared to the Without CL group (19.3%).

The results obtained in the present study could be associated, in addition to the presence of the CL, with luteal activity, since the larger size of the CL has been associated with high P4 production. The size of the CL and the concentration of progesterone are influenced by the day of the estrous cycle [18]. In a study involving sheep, estrous cycle monitoring by ultrasonography showed that the size of the CL was less than 100 mm^2^ during the first three days of the cycle; then, it increased linearly until day 12 (154.6 ± 11.8 mm^2^) and, finally, progressively decreased to 29.2 ± 5.3 mm^2^ on day 0. Similarly, the P4 concentration reached its maximum value (2.8 ± 0.5 ng mL^−1^) on day 12 [17] (González De Bulnes et al., 2000). Thus, the authors demonstrated a significant correlation between the area of the CL and the plasma concentration of P4 throughout the entire estrous cycle in sheep. In addition, in the ovaries of cows with CL, the concentration of P4 in the follicular fluid increased as the follicular diameter increased [30]. The local effects of P4 on the follicles remain poorly understood. A direct association has been proposed between the high concentration of P4 in the follicles ipsilateral to the CL and its interaction with the cumulus–oocyte complexes, with possible consequences in the acquisition of oocyte competence through the retraction of transzonal projections [14,31]. Furthermore, it has been shown that the P4 produced by the CL can diffuse to adjacent follicles, affecting embryo development [12].

Intracellular P4 signaling is mediated by its interaction with nuclear and membrane receptors in the COC, and it may be important for oocyte competence [32]. Furthermore, P4 prevents follicular and oocyte atresia [33], since an increase in LH promotes the expression of receptors in granulosa cells, favoring resistance to apoptosis [15] and maturation, thereby promoting nuclear formation [33]. In addition to the possible effects of P4 on oocyte competence [14], some authors have reported that ovaries with CL are almost twice the size of those without CL [4]. Therefore, a local effect has been suggested that is related to the high vascularization of the ovary, which allows for an increase in the flow of nutrients, hormones, and growth factors that favor follicular growth and oocyte development through intraovarian mechanisms [13,16].

Although hormone concentrations were not determined in the present study, the results support the positive effects of the presence and size of the CL, as they favor oocyte competence and subsequent in vitro embryo development until the blastocyst stage. However, no differences were observed in blastocyst size or hatching percentage. One of the limitations of this study was that the collected ovaries were mixed, so the effect of the CL on the contralateral ovary of the same sheep was not controlled or studied. Further research is needed to understand the mechanism by which this competence is improved and, based on the knowledge presented, to develop strategies to optimize the efficiency of in vitro-produced embryos, considering the importance of oocyte origin.

## 5. Conclusions

The size and presence of the corpus luteum in the ovaries of slaughtered sheep improved the number of follicles, the number and size of collected COCs, and the percentage of morula and blastocyst-stage embryos compared to ovaries without corpus luteum.

## Figures and Tables

**Table 1:** Influence of the absence or presence of CL (Small, Medium, or Large) in the ovaries of slaughterhouse sheep on the size of cumulus–oocyte complexes (COCs).

Group	Diameter of COCs, µm (LSM ± SE) *	COCs Area, µm^2^ (LSM ± SE) *
Without CL	248.5 ± 8.83 ^a^	49,175 ± 4136.40 ^a^
Small CL (≤3 mm)	285.8 ± 10.56 ^b^	66,560 ± 4943.94 ^b^
Medium CL (4–8 mm)	254.0 ± 9.58 ^ab^	51,325 ± 4486.55 ^ab^
Large CL (>8 mm)	272.2 ± 8.83 ^ab^	59,259 ± 4136.40 ^ab^

LSM, least-squares mean; SE, standard error. * LSM ± SE, in the same column with different letters are different (*p* < 0.05).

**Table 2:** Influence of the absence or presence of CL (Small, Medium, or Large) in the ovaries of slaughterhouse sheep on the in vitro production of morulae.

Group	Number of COCs	Number of Morulae	Morulae (LSM ± SE, %) *
Without CL	339	264	78.59 ± 3.13 ^a^
Small CL (≤3 mm)	274	224	81.74 ± 2.58 ^ab^
Medium CL (4–8 mm)	197	168	85.30 ± 3.53 ^ab^
Large CL (>8 mm)	185	162	88.01 ± 3.70 ^b^

COCs, cumulus–oocyte complexes; LSM, least-squares mean; SE, standard error. * LSM ± SE, % in the same column with different letters are different (*p* < 0.05).

**Table 3:** Influence of the absence or presence of CL (Small, Medium, or Large) in the ovaries of slaughterhouse sheep on the in vitro production of blastocysts.

Group	Number of Oocytes	Number of Blastocysts	Blastocysts (LSM ± SE, %) *	Hatching Capacity (LSM ± SE, %) *
Without CL	339	132	39.48 ± 3.64 ^a^	22.41 ± 5.41 ^a^
Small CL (≤3 mm)	274	123	44.89 ± 3.29 ^a^	20.22 ± 4.23 ^a^
Medium CL (4–8 mm)	197	102	52.63 ± 5.48 ^b^	19.61 ± 5.52 ^a^
Large CL (>8 mm)	185	94	51.19 ± 5.63 ^ab^	24.39 ± 6.76 ^a^

LSM, least-squares mean; SE, standard error. * LSM ± SE, % in the same column with different letters are different (*p* < 0.05).

**Table 4:** Influence of the absence or presence of CL (Small, Medium, or Large) in the ovaries of slaughter sheep on the diameter of blastocysts produced in vitro.

Group	Blastocysts, *n*	Blastocyst Diameter (LSM ± SE, µm)
Without CL	69	211.7 ± 6.40
Small CL (≤3 mm)	59	212.3 ± 4.48
Medium CL (4–8 mm)	60	207.6 ± 6.29
Large CL (>8 mm)	51	202.0 ± 7.52

LSM, least-squares mean; SE, standard error.

## Data Availability

The raw data supporting the conclusions of this article will be made available by the authors on request.
